# Synergism and Antagonism of Bacterial-Viral Coinfection in the Upper Respiratory Tract

**DOI:** 10.1128/msphere.00984-21

**Published:** 2022-01-19

**Authors:** Sam Manna, Julie McAuley, Jonathan Jacobson, Cattram D. Nguyen, Md. Ashik Ullah, Ismail Sebina, Victoria Williamson, E. Kim Mulholland, Odilia Wijburg, Simon Phipps, Catherine Satzke

**Affiliations:** a Infection and Immunity, Murdoch Children’s Research Institute, Royal Children's Hospital, Parkville, Victoria, Australia; b Department of Paediatrics, The University of Melbourne, Parkville, Victoria, Australia; c Department of Microbiology and Immunology at the Peter Doherty Institute for Infection and Immunity, The University of Melbourne, Melbourne, Victoria, Australia; d Respiratory Immunology Laboratory, QIMR Berghofer Medical Research Institute, Herston, Queensland, Australia; e Department of Infectious Disease Epidemiology, London School of Hygiene and Tropical Medicine, London, United Kingdom; National Institute of Allergy and Infectious Diseases

**Keywords:** *Streptococcus pneumoniae*, pneumococcus, respiratory syncytial virus, pneumonia virus of mice, murine pneumonia virus, influenza, coinfection

## Abstract

Streptococcus pneumoniae (the pneumococcus) is a leading cause of pneumonia in children under 5 years of age. Coinfection by pneumococci and respiratory viruses enhances disease severity. Little is known about pneumococcal coinfections with respiratory syncytial virus (RSV). Here, we developed a novel infant mouse model of coinfection using pneumonia virus of mice (PVM), a murine analogue of RSV, to examine the dynamics of coinfection in the upper respiratory tract, an anatomical niche that is essential for host-to-host transmission and progression to disease. Coinfection increased damage to the nasal tissue and increased production of the chemokine CCL3. Nasopharyngeal pneumococcal density and shedding in nasal secretions were increased by coinfection. In contrast, coinfection reduced PVM loads in the nasopharynx, an effect that was independent of pneumococcal strain and the order of infection. We showed that this “antagonistic” effect was absent using either ethanol-killed pneumococci or a pneumococcal mutant deficient in capsule production and incapable of nasopharyngeal carriage. Colonization with a pneumococcal strain naturally unable to produce capsule also reduced viral loads. The pneumococcus-mediated reduction in PVM loads was caused by accelerated viral clearance from the nasopharynx. Although these synergistic and antagonistic effects occurred with both wild-type pneumococcal strains used in this study, the magnitude of the effects was strain dependent. Lastly, we showed that pneumococci can also antagonize influenza virus. Taken together, our study has uncovered multiple novel facets of bacterial-viral coinfection. Our findings have important public health implications, including for bacterial and viral vaccination strategies in young children.

**IMPORTANCE** Respiratory bacterial-viral coinfections (such as pneumococci and influenza virus) are often synergistic, resulting in enhanced disease severity. Although colonization of the nasopharynx is the precursor to disease and transmission, little is known about bacterial-viral interactions that occur within this niche. In this study, we developed a novel mouse model to examine pneumococcal-viral interactions in the nasopharynx with pneumonia virus of mice (PVM) and influenza. We found that PVM infection benefits pneumococci by increasing their numbers in the nasopharynx and shedding of these bacteria in respiratory secretions. In contrast, we discovered that pneumococci decrease PVM numbers by accelerating viral clearance. We also report a similar effect of pneumococci on influenza. By showing that coinfections lead to both synergistic and antagonistic outcomes, our findings challenge the existing dogma in the field. Our work has important applications and implications for bacterial and viral vaccines that target these microbes.

## INTRODUCTION

The severity and outcome of microbial infections are determined by a variety of host, pathogen, and environmental factors. As the pathogen colonizes the host, it encounters members of the resident microbiota and/or other pathogens. These interactions can influence microbial pathogenesis, including increased bacterial adhesion, enhanced virion stability, and modulation of the immune response by one microbe that benefits the other (1). These interactions can be particularly relevant in anatomical sites that have complex microbial communities, including the gastrointestinal and respiratory tracts (2–6).

Pneumococcus is a leading cause of community-acquired pneumonia in young children, particularly for those in low- and middle-income settings (7, 8). This bacterium is a common resident of the upper respiratory tract in children, with carriage prevalence declining with age (9). While nasopharyngeal colonization by pneumococci is usually asymptomatic, it serves as a prerequisite for disease and a reservoir for host-to-host transmission, which underpins herd protection conferred by pneumococcal conjugate vaccines (10, 11). Throughout history, 30 to 95% of severe or fatal cases during influenza pandemics were caused by secondary bacterial infections, with pneumococci being the leading etiologic agent (12). In part, this is because the immune response to viral infection creates a hospitable environment for pneumococcal superinfection (12). Influenza infection also has positive effects on pneumococci in the upper respiratory tract, exemplified by increased pneumococcal density in the nasopharynx of mice (13, 14), as well as increased transmission to naive hosts, which has been demonstrated in both animal and human studies (13, 15, 16).

Human respiratory syncytial virus (RSV) disproportionately affects infants and is a major cause of bronchiolitis and infant hospitalization (2). Although little is known regarding the mechanisms of pneumococcal-RSV coinfection, there is clear evidence of its clinical relevance. Detection of both RSV and pneumococcus in young children under 2 years of age presenting to a hospital with acute respiratory infection is associated with increased disease severity (17). Pneumococcal infection in preterm infants hospitalized with RSV is associated with longer hospital stays and an increased likelihood of admission to the intensive care unit (18). Importantly, introduction of pneumococcal conjugate vaccination has been associated with a decline in RSV hospitalizations in children (19, 20). Lastly, direct interactions between the pneumococcal penicillin-binding protein 1a and RSV G protein enhance pneumococcal virulence in mice (21).

For this study, we developed an animal model to assess the dynamics of pneumococcal-RSV coinfection in the upper respiratory tract. Interactions between pneumococci and RSV in the upper respiratory tract are poorly understood, but this site plays an important role in the pathogenesis of both microbes. We opted to develop an infant mouse model, as it offers several advantages over the use of adult mice. In humans, pneumococcal carriage prevalence declines with age (9) and the immune system of children is somewhat underdeveloped (22). Compared with adult mice, infant mice are more susceptible to pneumococcal carriage, so a lower, more realistic dose can be used to produce stable, high-density colonization for a longer duration (13, 14, 23). Additionally, although development of the immune system in mice is not identical to that of humans, infant mice are born with an immature immune system that develops over time, similar to that observed in human infants (24). Lastly, by having multiple pups in the same litter in close contact, the infant mouse model offers the ability to examine pneumococcal transmission between littermates (13).

Recent work has shown that most infants are already colonized by pneumococci at the time that they contract RSV (25). Additionally, children in low- and middle-income settings are often colonized by pneumococci early in life (8) and are therefore also likely to carry pneumococci at the time of viral infection. To represent coinfections in these high-risk settings, we established a murine model where pneumococci colonize the nasopharynx before viral challenge. Human RSV replicates poorly in mice. Existing models therefore rely on administering high viral titers (∼10^4^ to 10^6^ PFU) (26–30), which are less likely to be biologically relevant. This is because the infectious dose to establish RSV infection in adult volunteers is as low as 10^2^ to 10^3^ and is anticipated to be even lower in infants (31–33). To overcome this, we used pneumonia virus of mice (PVM; also known as murine pneumonia virus), a murine analogue of RSV (34, 35) and member of the *Orthopneumovirus* genus. PVM replicates in the airway epithelium and induces pathological features in mice similar to those observed in humans with RSV disease (36, 37). By challenging pneumococcus-colonized infant mice with PVM, we were able to develop a novel pneumococcal-PVM model and elucidate the host-, virus-, and bacterium-dependent effects of coinfection in the upper respiratory tract.

## RESULTS

### Development of an infant mouse model of pneumococcal-PVM coinfection.

**FIG 1:**
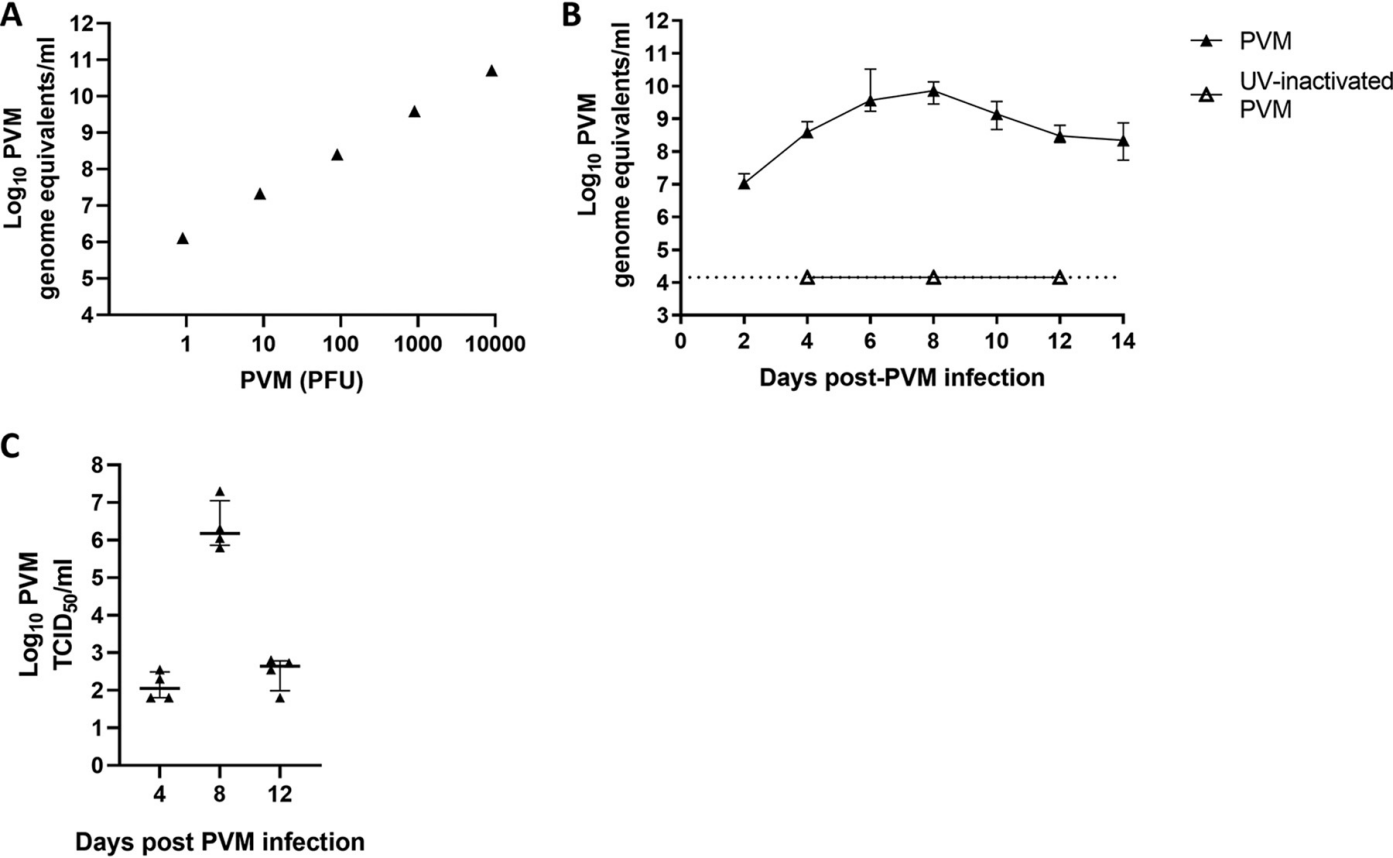
Establishing a PVM detection assay and upper respiratory tract infection in mice. (A) Correlation of a PVM qRT-PCR assay with infectious virus. (B) Kinetics of live PVM and UV-inactivated PVM in the nasopharynx of infant C57BL/6 mice as determined by qRT-PCR. Mice were infected intranasally with 10 PFU (7.33 log_10_ genome equivalents/ml) at 9 days of age. The black dotted line represents the qRT-PCR limitation of detection. (C) Detection of infectious PVM from mice 4, 8, and 12 days post-PVM infection by TCID_50_. Data are medians ± IQR (*n* = 8 to 10 mice per group).

Given that PVM loads from mouse tissues are difficult to quantify by plaque assay (38) and our requirement to test a large number of mouse samples, we employed quantitative reverse transcription-PCR (qRT-PCR) as a high-throughput method to detect and quantify PVM. As qRT-PCR does not differentiate between infectious and noninfectious viruses, we first validated the qRT-PCR assay by running a dilution series of infectious stocks containing known quantities of infectious PVM determined by plaque assay. Although numbers of genome copies determined by qRT-PCR assay overestimate viral loads, they correlate with levels of infectious virus (Fig. 1A) (*P* = 0.008, *r* = 1.00, Spearman’s correlation) and are therefore appropriate to identify differences in viral loads between groups.

To determine whether PVM infection could be established in the upper respiratory tract of infant mice, 9-day-old C57BL/6 pups were given 10 PFU of PVM via intranasal administration without anesthesia (equivalent to 7.33 log_10_ PVM genome equivalents/ml). Previously, we have shown that this dose induces a largely asymptomatic infection of the lungs when delivered by intranasal administration under anesthesia (39, 40). Viral replication in the upper respiratory tract was measured using qRT-PCR and peaked at 9.86 (interquartile range [IQR], 9.23 to 10.73) log_10_ PVM genome equivalents/ml at 8 days postinfection (Fig. 1B). When we infected 9-day-old mice with 10 PFU of UV-inactivated PVM, we were unable to detect any PVM genome copies in the nasopharynx of these mice (Fig. 1B), despite PVM remaining detectable in the initial inoculum. In further support of PVM replication in the upper respiratory tract, we confirmed that infectious virus could be recovered from mouse nasopharyngeal tissue homogenates by determining the 50% tissue culture infective dose (TCID_50_) (Fig. 1C), with higher TCID_50_ values in mouse tissues collected at 8 days than at 4 days post-PVM infection, before decreasing at 12 days post-PVM infection. These TCID_50_ data were consistent and correlated with our qRT-PCR assay (*r* = 0.81, *P* = 0.002, Spearman's correlation).

**FIG 2:**
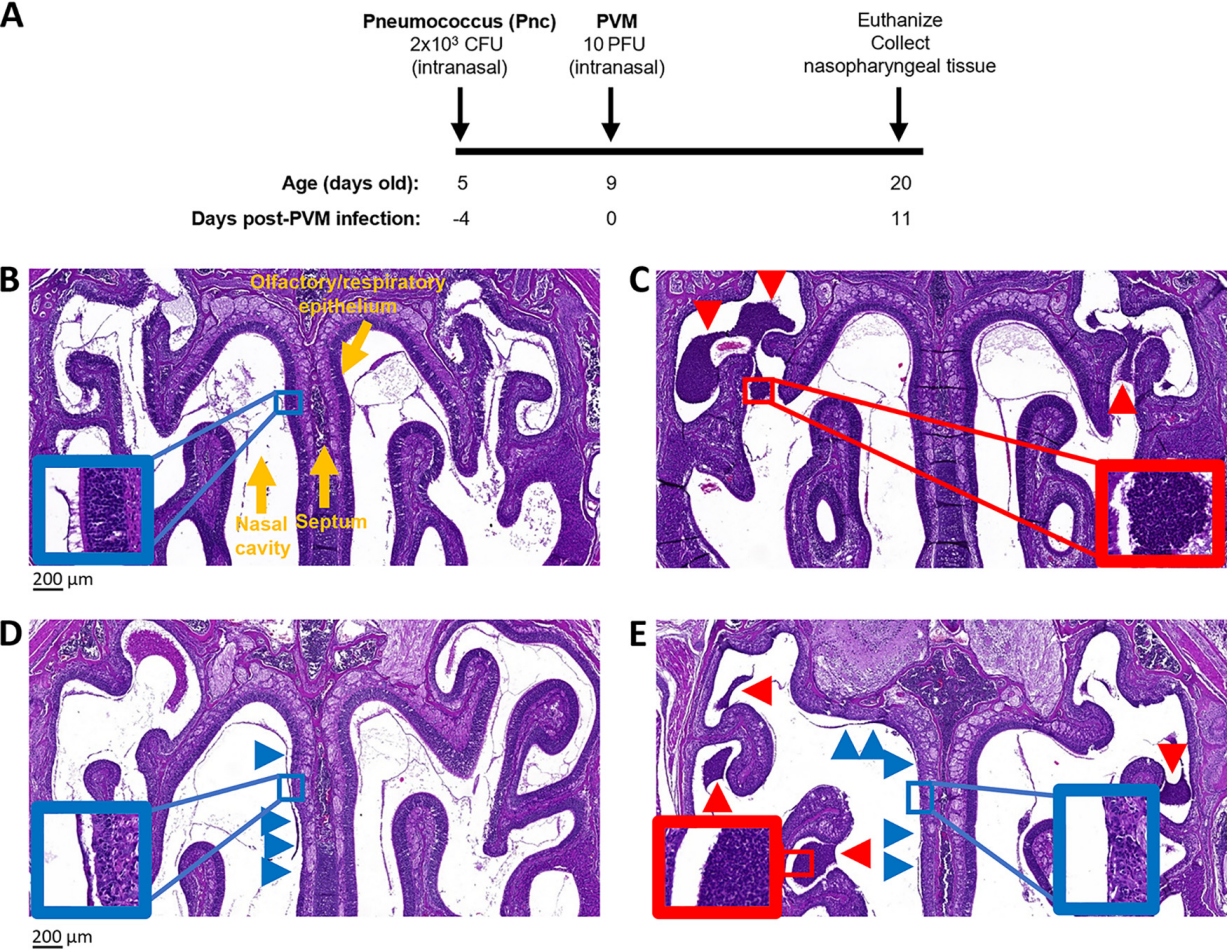
Pneumococcal-PVM coinfection model. (A) Schematic of pneumococcal-PVM coinfection model. PBS was administered as a vehicle control. All experiments follow this model of primary pneumococcal and secondary PVM administration (unless otherwise stated). (B to E) Representative images of hematoxylin and eosin stains of nasal sections (5 μm thick). Sections were taken from 20-day-old mice that had been administered PBS (mock) (B), pneumococci only (C), or PVM only (D) or were coinfected (E) (*n* = 4 mice per group). Images were taken using a ×5 magnification lens. Scale bar = 200 μm. Insets (denoted by boxes) are at a ×20 magnification. Blue arrowheads and boxes highlight areas of necrosis along the respiratory epithelium. Red arrowheads and boxes highlight inflammatory cells.

After demonstrating PVM replication in the upper respiratory tract, we adapted our model to incorporate pneumococcal coinfection. Two pneumococcal strains were used in this study: EF3030 and BCH19. Both of these are clinical isolates of the same capsular serotype (19F). For our coinfection model (Fig. 2A), mice were given 2 × 10^3^ CFU of pneumococci by intranasal administration at 5 days of age, followed 4 days later with PVM infection. Neither mono- nor coinfected mice exhibited any clinical signs of disease, and both groups survived to the experimental endpoint without any health or welfare issues.

Histopathological analysis of nasal sections found that mock-infected mice had no observable changes (median histopathology score of 0) (Fig. 2B). In contrast, mice that received pneumococci alone exhibited submucosal inflammation of the respiratory epithelium and inflammatory cells (median histopathology score of 3.94 [IQR, 3.16 to 5.47]) (Fig. 2C). Mice infected with PVM alone had mild submucosal inflammation, as well as unilateral olfactory sensory neuronal necrosis along the respiratory epithelium (median histopathology score of 4.5 [IQR, 1.56 to 5.84]) (Fig. 2D). Coinfected mice exhibited the combined effects of inflammatory cells in the nasal cavity and sensory neuronal necrosis (median histopathology score of 8.19 [IQR, 7.13 to 9.19]) (Fig. 2E). Coinfected mice had the highest histopathological grading for tissue damage and inflammation compared with the scores of all other groups (*P* = 0.029 for mock-, pneumococcus-, and PVM-infected groups; Mann-Whitney test). Using the Bliss independence test (41), we found that the effects observed in the nasal tissues of coinfected mice were synergistic rather than additive (Bliss independence score of 0.25).

**FIG 3:**
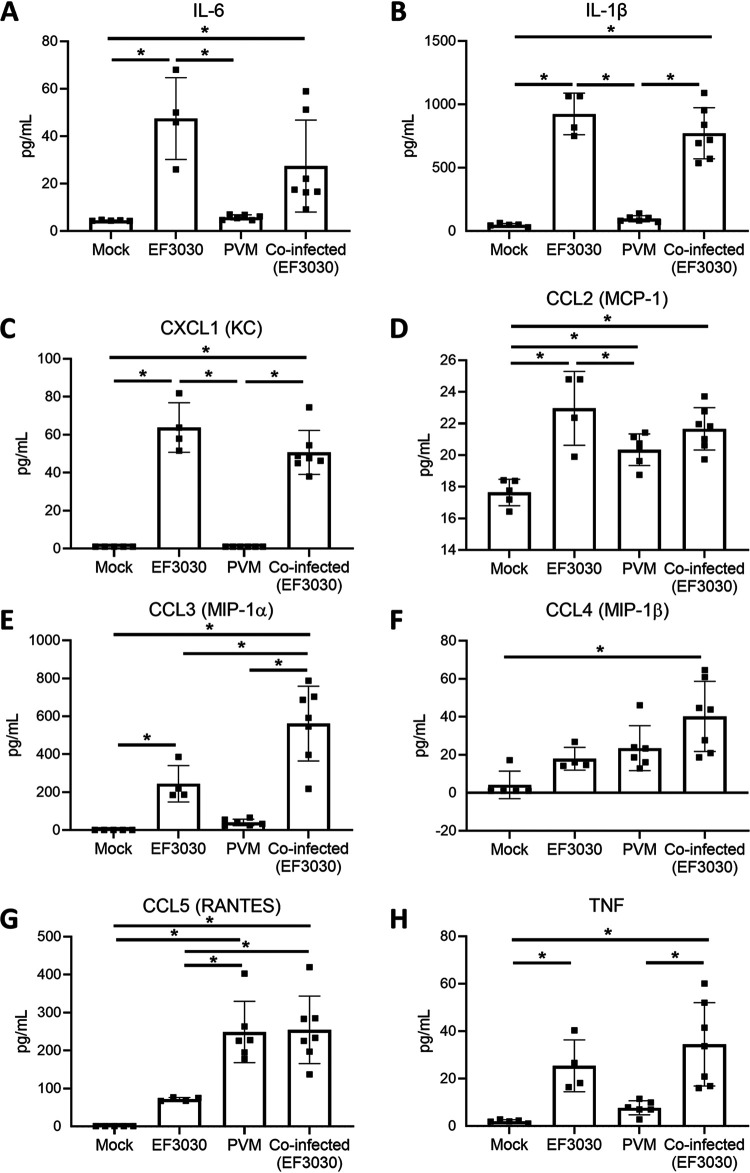
Proinflammatory cytokine levels during pneumococcal-PVM coinfection. (A to H) Concentrations of proinflammatory cytokines in nasopharyngeal homogenates of 20-day-old mice that were mock infected, given pneumococci (EF3030) only, given PVM only, or coinfected. Data are presented as means ± standard deviations and were analyzed by one-way ANOVA (*n* = 4 to 7 mice per group). Only *P* values of <0.05 (*) are shown.

We next analyzed the inflammatory chemokine and cytokine responses in nasopharyngeal tissue. Assessment of proinflammatory cytokines present in the supernatants of homogenized samples of nasopharyngeal tissue revealed that mice colonized with pneumococci (EF3030 strain) had higher concentrations of interleukin 6 (IL-6), IL-1β, CXCL1 (keratinocyte-derived chemokine [KC]), CCL2, CCL3, and tumor necrosis factor (TNF) than the mock-infected control (*P* < 0.001 for IL-6, IL-1β, CXCL1, and CCL2, and *P* = 0.03 for CCL3 and TNF; one-way analysis of variance [ANOVA]) (Fig. 3). Mice infected with PVM alone exhibited elevated levels of CCL2 and CCL5 compared with levels in the mock-infected control (*P* = 0.02 and < 0.001, respectively; one-way ANOVA). All chemokines and cytokines detected in the tissues of coinfected mice were elevated compared with levels in mock-infected mice (IL-6, *P* = 0.04; all other chemokines and cytokines, *P* < 0.001; one-way ANOVA). Of note, CCL3 concentrations were increased during coinfection compared with concentrations in other mono-infected mice (*P* = 0.003 and < 0.001 for mice infected with pneumococci only and PVM only, respectively) and mock-infected experimental groups (*P* < 0.001) (Fig. 3E). This was a synergistic rather than an additive effect (Bliss independence score of 0.28).

### Effects of coinfection on pneumococci.

Inflammation induced by influenza virus increases pneumococcal density (13), and nasopharyngeal samples from children with RSV have higher pneumococcal loads than children without RSV (42, 43). Therefore, we examined the density of pneumococci in the nasopharynx of mice, with and without PVM administration. At 20 days old (11 days post-PVM infection), coinfection increased the median nasopharyngeal density of BCH19 ∼10-fold (5.30 [IQR, 5.21 to 5.50] and 6.29 [IQR, 5.85 to 6.48] log_10_ CFU per nasopharynx for mice administered BCH19 alone and coinfected mice, respectively; *P* < 0.0001, Mann-Whitney test).

**FIG 4:**
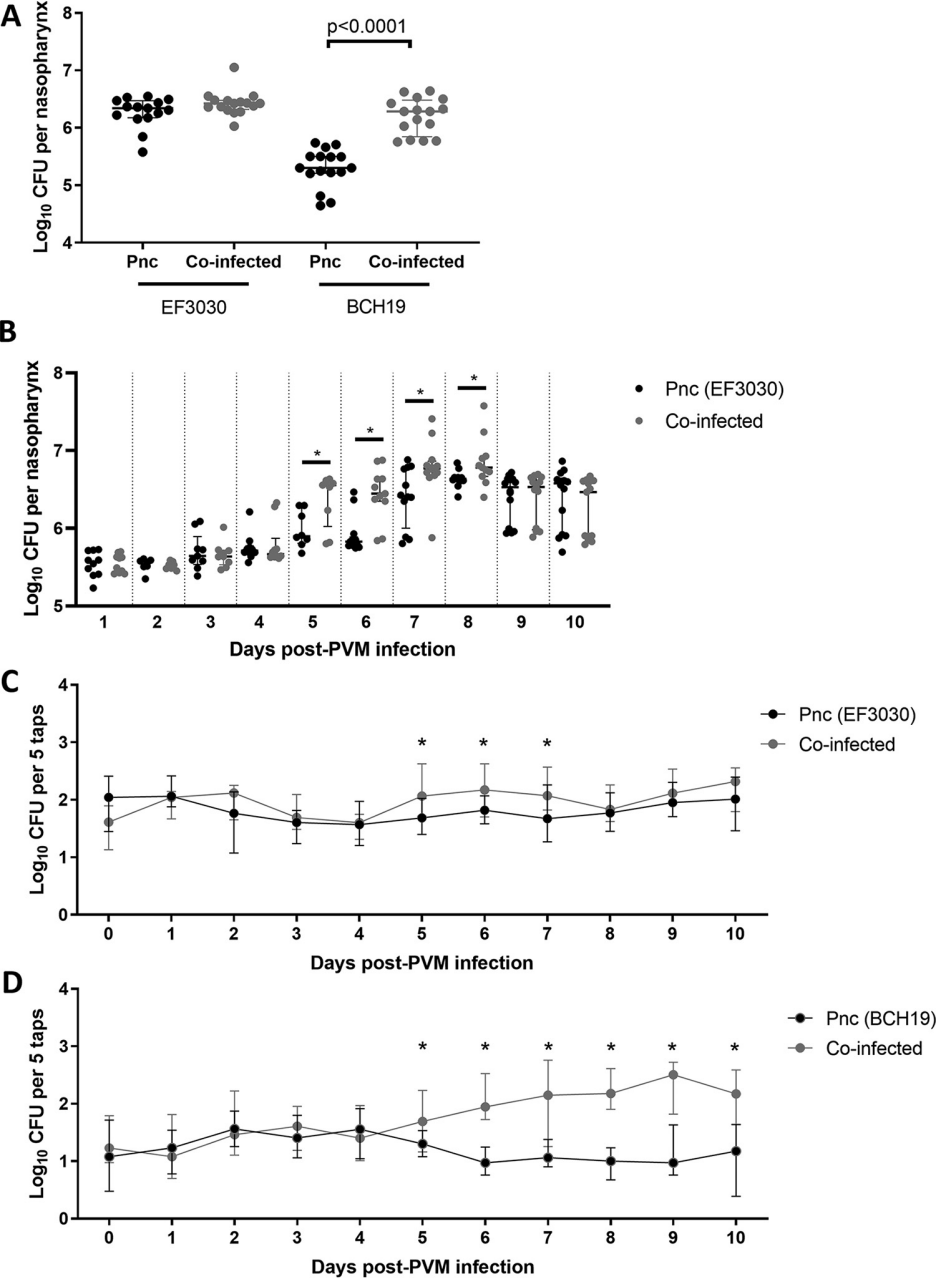
Effect of coinfection on pneumococci. (A) Nasopharyngeal densities of pneumococcal strains EF3030 and BCH19 in 20-day-old (11 days post-PVM infection) mice given pneumococci alone (Pnc) or coinfected with PVM (∼15 mice per group). (B) Nasopharyngeal density of EF3030 in mice given pneumococci alone (Pnc) or coinfected with PVM over time (*n* = 10 to 14 mice per group). (C and D) Shedding of pneumococcal strains EF3030 (C) and BCH19 (D) in the nasal secretions of coinfected mice, compared with those given pneumococci alone (Pnc) (*n* = 15 to 20 mice per group). Data are presented as medians ± IQR and were analyzed by the Mann-Whitney test. Only *P* values of <0.05 (*) are shown.

PVM had no effect on the density of EF3030 at the same time point (6.34 [IQR, 6.18 to 6.47] and 6.43 [IQR, 6.32 to 6.48] log_10_ CFU per nasopharynx for mice administered EF3030 alone and coinfected mice, respectively; *P* = 0.137, Mann-Whitney test) (Fig. 4A). However, when we examined EF3030 density over time, we found that coinfection increased the median nasopharyngeal density of EF3030 by approximately 0.66, 0.62, 0.36 and 0.14 log_10_ from 5 to 8 days post-PVM infection (14 to 17 days old) (*P* = 0.033, 0.0004, 0.032, and 0.032, respectively; Mann-Whitney test) (Fig. 4B). This PVM-mediated increase in EF3030 density was not observed from 9 days post-PVM infection onwards.

Given that influenza-induced inflammation increases pneumococcal transmission (13, 15), we postulated that PVM infection may have similar effects on pneumococcus-colonized mice. Pneumococcal transmission involves shedding of bacteria from a colonized individual (egress), survival on fomites, and subsequently entering and colonizing a new host (acquisition) (11). To measure egress from a colonized host, we quantified pneumococci within nasal secretions by tapping the nares of each mouse on selective media to culture pneumococci. Compared with mice colonized with pneumococci alone, mice coinfected with PVM had higher numbers of pneumococci shed in their nasal secretions from 5 days post-PVM infection (Fig. 4C and D). Interestingly, we found that the duration and magnitude of the increase in pneumococcal shedding were dependent on the bacterial strain. PVM increased EF3030 shedding by approximately 0.36 log_10_ from 5 to 7 days post-PVM infection (*P* = 0.006, 0.014, and 0.022, respectively; Mann-Whitney test) (Fig. 4C). For BCH19-colonized mice, PVM increased shedding by approximately 1.04 log_10_ from 5 to 10 days post-PVM infection (*P* = 0.036, < 0.0001, 0.002, < 0.0001, < 0.0001, and 0.0002, respectively; Mann-Whitney test) (Fig. 4D).

To investigate acquisition, we adapted our previous pneumococcal transmission model (13), replacing influenza virus with PVM. We used the same timeline as described for Fig. 2A; however, only half of each litter of mice was administered pneumococci (index mice), while the other half remained uninfected (contact mice). We then administered PVM to both index and contact mice and assessed pneumococcal colonization in contact mice 11 days post-PVM infection. This experimental design meant that PVM replication would peak in the middle of the “transmission window,” providing sufficient time for PVM to facilitate pneumococcal transmission. For BCH19, there was no difference in the proportions of contact mice acquiring pneumococci when mice were coinfected with PVM (4/14, 29%) or given pneumococci alone (4/15, 27%) (*P* > 0.999, Fisher’s exact test). For EF3030, 6/15 (40%) contact mice acquired pneumococci when they were coinfected, compared with 2/14 (14%) mice given pneumococci alone. However, this did not reach statistical significance (*P* = 0.215, Fisher’s exact test).

### Effects of coinfection on PVM.

**FIG 5:**
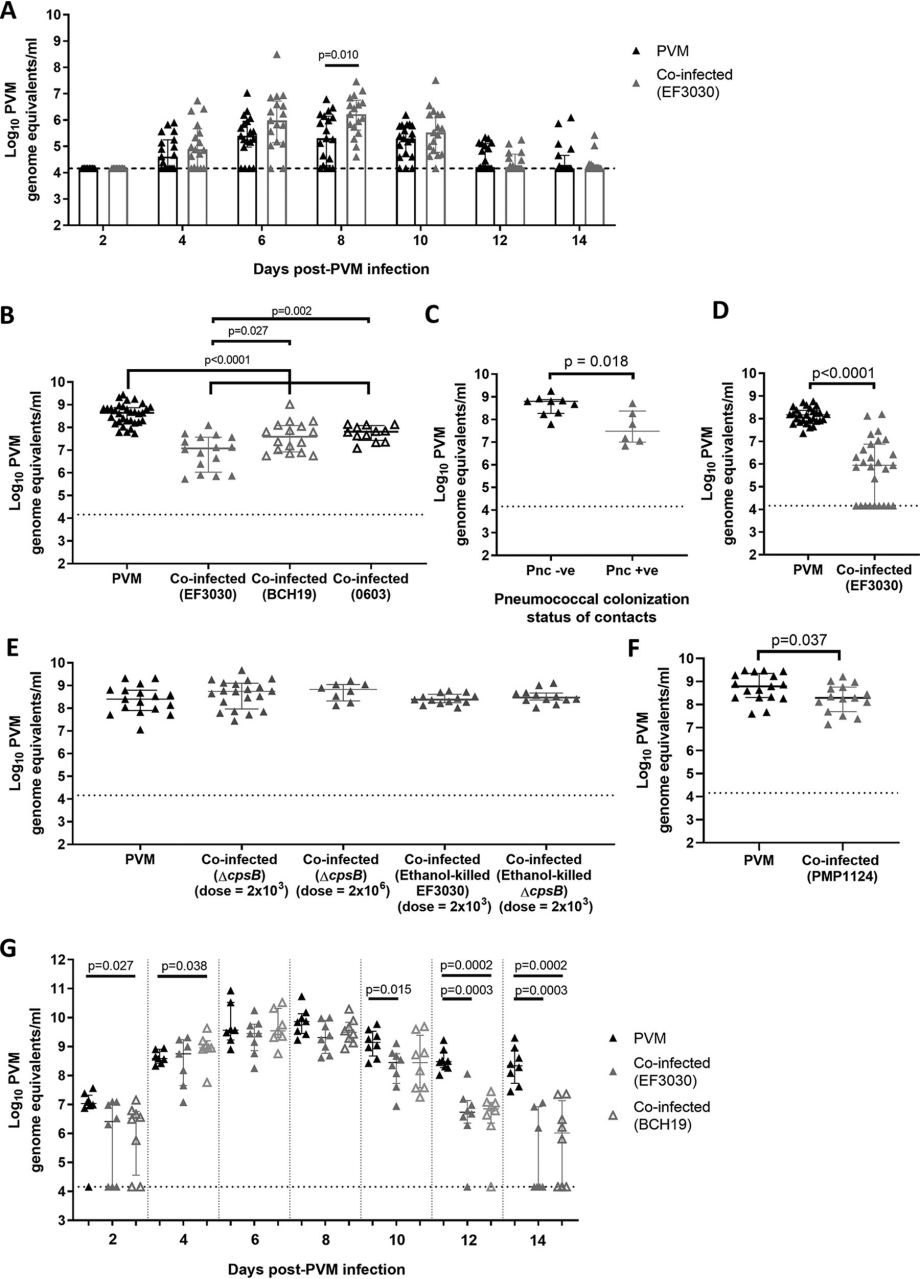
Effect of coinfection on PVM. (A) Shedding of PVM in nasal secretions of mice coinfected with pneumococcal strain EF3030 or given PVM alone (*n* = 16 to 19 mice per group). Viral loads were determined by qRT-PCR. (B) PVM loads in 20-day-old mice coinfected with pneumococci (strain EF3030, BCH19, or 0603) or given PVM alone (*n* = 12 to 16 mice per group; 33 for the PVM control group). (C) PVM loads in the nasopharynx of 20-day-old contact mice that naturally acquired pneumococci (pneumococcus positive [Pnc +ve]) or did not acquire pneumococci (pneumococcus negative [Pnc –ve]) from their coinfected littermates (*n* = 6 to 9 mice per group). Half of the litter of 5-day-old pups were given pneumococcal strain EF3030 (index mice), while the other half (contacts) were not. At 9 days of age, index and contact mice were given PVM. Nasopharyngeal tissue was harvested from 20-day-old euthanized pups for quantification of PVM by qRT-PCR, as well as for pneumococcal culture. (D) Effect of secondary pneumococcal administration on PVM loads in 20-day-old mice (*n* = 27 to 28 mice per group). Mice were given PVM at 5 days of age, followed by pneumococcal administration at 9 days of age. (E) PVM loads in the nasopharynx of 20-day-old mice that had been coinfected by pneumococcal colonization/capsule Δ*cpsB* mutant at two doses (2 × 10^3^ and 2 × 10^6^ CFU) or ethanol-killed pneumococci. Mice were infected using the original timeline (pneumococci and then PVM at 5 and 9 days of age, respectively) (*n* = 8 to 20 mice per group). (F) PVM loads in the nasopharynx of 20-day-old mice coinfected with the nonencapsulated, colonizing strain PMP1124. (G) PVM loads over time in mice coinfected with pneumococci (EF3030 or BCH19), compared with loads in mice given PVM alone (*n* = 7 to 8 mice per group). The black dotted line represents the qRT-PCR limitation of detection. In the qRT-PCR assay, 10 PFU of PVM is equivalent to 7.33 log_10_ genome equivalents/ml. Data are presented as medians ± IQR and were analyzed by the Mann-Whitney test. Only *P* values of <0.05 are shown.

We next examined how pneumococcal colonization affects PVM. Using the same timeline for coinfection (Fig. 2A), we measured shedding of PVM by swabbing the anterior nares of mice for viral quantification by qRT-PCR every 2 days post-PVM administration. Mice carrying pneumococci (EF3030 strain) exhibited a 0.91 log_10_ increase in median viral shedding at 8 days post-PVM infection (6.22 [IQR, 5.62 to 6.75] log_10_ PVM genome equivalents/ml) compared with shedding in mice given PVM alone (5.31 [IQR, 4.27 to 6.15]; *P* = 0.010, Mann-Whitney test) (Fig. 5A). This corresponds to the period of peak viral load (Fig. 1B). Additionally, a trend toward increased shedding at 6 days post-PVM infection occurred, with a 0.59 log_10_ increase in shedding detected in coinfected mice (5.99 [IQR, 5.14 to 6.72] log_10_ PVM genome equivalents/ml) compared with that of mice administered PVM alone (5.40 [IQR, 4.97 to 5.95] log_10_ PVM genome equivalents/ml) (*P* = 0.085, Mann-Whitney test).

Unexpectedly, PVM loads in the nasopharynx of coinfected mice were lower than those in mice that had received PVM alone (Fig. 5B). At 11 days post-PVM infection, mice colonized with EF3030 had a 1.63 log_10_ reduction in PVM loads (7.01 [IQR, 6.03 to 7.57] log_10_ PVM genome equivalents/ml) compared with those of mice given PVM alone (8.64 [IQR, 8.25 to 8.87]; *P* < 0.0001, Mann-Whitney test). Mice colonized with BCH19 had a 1.05 log_10_ reduction in PVM loads (7.59 [IQR, 6.92 to 8.08] log_10_ PVM genome equivalents/ml) compared with loads in mice given PVM alone (*P* < 0.0001, Mann-Whitney test). As both EF3030 and BCH19 are serotype 19F, we tested a third pneumococcal strain (0603, serotype 6B), finding that it also caused a reduction in viral loads, as there was a 0.84 log_10_ reduction in PVM loads in coinfected mice (7.81 [IQR, 7.43 to 8.08] log_10_ PVM genome equivalents/ml) compared with those in mice given PVM alone (*P* < 0.0001, Mann-Whitney test) (Fig. 5B).

In the transmission experiment described above, 40% of PVM-infected contact mice acquired EF3030 from their index littermates. Interestingly, when we examined PVM loads in contact mice, those that had acquired EF3030 (from their littermates) had reduced PVM loads (7.45 [IQR, 7.00 to 8.38] log_10_ PVM genome equivalents/ml) compared with those of contacts that were not colonized with EF3030 (8.81 [IQR, 8.27 to 8.88]; *P* = 0.018, Mann-Whitney test) (Fig. 5C). Therefore, we hypothesized that pneumococcus-mediated antagonism of PVM may also occur when pneumococci are acquired after viral infection. To test this, we reversed the order of infection in our model by administering PVM at 5 days of age, followed by pneumococci at 9 days of age. We found that the secondary pneumococcal administration also induced a reduction (2.2 log_10_) in PVM loads (5.95 [IQR, 4.16 to 6.88] log_10_ PVM genome equivalents/ml) compared with loads in mice given PVM alone (8.17 [IQR, 7.90 to 8.37]; *P* < 0.0001, Mann-Whitney test) (Fig. 5D).

To examine the contribution of bacterial factors to the reduction of PVM loads, we generated a pneumococcal strain deficient in the ability to produce capsular polysaccharide, by deleting *cpsB* (also known as *wzh*), one of the major regulatory genes of polysaccharide synthesis. Δ*cpsB* strains produce less capsule (44, 45), and this gene is important for nasopharyngeal carriage in mice (46). Consistently with this, we were unable to recover any Δ*cpsB* mutants from the nasopharynx of 20-day-old mice (*n* = 16 mice). In contrast, the EF3030 parent strain colonized the nasopharynx of all mice at the same time point (Fig. 4A). In our coinfection model, the Δ*cpsB* mutant did not reduce PVM loads (8.75 [IQR, 7.97 to 9.10] log_10_ PVM genome equivalents/ml) compared with those of mice given PVM alone (8.40 [IQR, 7.90 to 8.80]; *P* = 0.270, Mann-Whitney test) (Fig. 5E). Increasing the dose of Δ*cpsB* by 1,000-fold did not restore the reduction in PVM loads (8.83 [IQR, 8.32 to 9.04]; *P* = 0.175, Mann-Whitney test) (Fig. 5E).

As the Δ*cpsB* mutant produces less capsule and is incapable of nasopharyngeal carriage, it was unclear whether the pneumococcus-mediated reduction in PVM loads required exposure to capsular polysaccharide (and/or other pattern recognition receptor ligands) or stable pneumococcal colonization of the nasopharynx. To help decipher this, we administered ethanol-killed EF3030 or the Δ*cpsB* mutant and examined PVM loads. Neither ethanol-killed EF3030 (8.39 [IQR, 8.25 to 8.61]; *P* = 0.967, Mann-Whitney test) (Fig. 5E) nor the Δ*cpsB* mutant (8.47 [IQR, 8.36 to 8.67]; *P* = 0.527, Mann-Whitney test) (Fig. 5E) exerted any effect on PVM loads in the nasopharynx compared with loads in mice given PVM alone.

To distinguish whether the pneumococcus-mediated reduction in PVM loads was due to exposure to pneumococcal capsule or from colonization, we tested PMP1124, a naturally occurring nonencapsulated pneumococcal strain that can colonize. Colonization was further supported by the recovery of PMP1124 from the nasopharynx of 20-day-old mice (4.24 [IQR, 3.71 to 4.59] log_10_ CFU per nasopharynx; *n* = 13 mice). Mice colonized with PMP1124 exhibited a 0.5 log_10_ reduction in PVM loads (8.29 [IQR, 7.69 to 8.75] log_10_ PVM genome equivalents/ml) compared with loads in mice given PVM alone (8.79 [IQR, 8.30 to 9.35]; *P* = 0.037, Mann-Whitney test) (Fig. 5F).

To investigate the reduction in PVM loads in mice colonized with pneumococci further, we evaluated viral loads over time. PVM was administered to 9-day-old mice 4 days after they had received pneumococci. Viral loads were then measured every 2 days post-PVM infection. There was a significant difference in PVM loads between mice colonized with BCH19 and control mice (PVM only) at 2 and 4 days post-PVM infection (*P* = 0.027 and 0.038, Mann-Whitney test), respectively (Fig. 5G). Otherwise, viral loads were similar between the control and coinfected mice for the first 8 days post-PVM infection (Fig. 5G). During the resolution phase (from 10 to 14 days post-PVM infection), all groups had a reduction in viral loads over time. Mice colonized with pneumococci had a greater reduction in PVM loads at each time point in the resolution phase than controls given PVM only (Fig. 5G). Additionally, the magnitude of the reduction in PVM loads in pneumococcus-colonized mice (compared with mice given PVM only) increased with each time point. There were 0.7, 1.75, and 4.19 log_10_ reductions in PVM loads in EF3030-colonized mice at 10, 12, and 14 days post-PVM infection (*P* = 0.015, 0.0003, and 0.0003, Mann-Whitney test), respectively. Similarly, for BCH19-colonized mice, there were 1.62 and 2.29 log_10_ reductions at 12 and 14 days post-PVM infection (*P* = 0.0002 for both time points, Mann-Whitney test), respectively.

**FIG 6:**
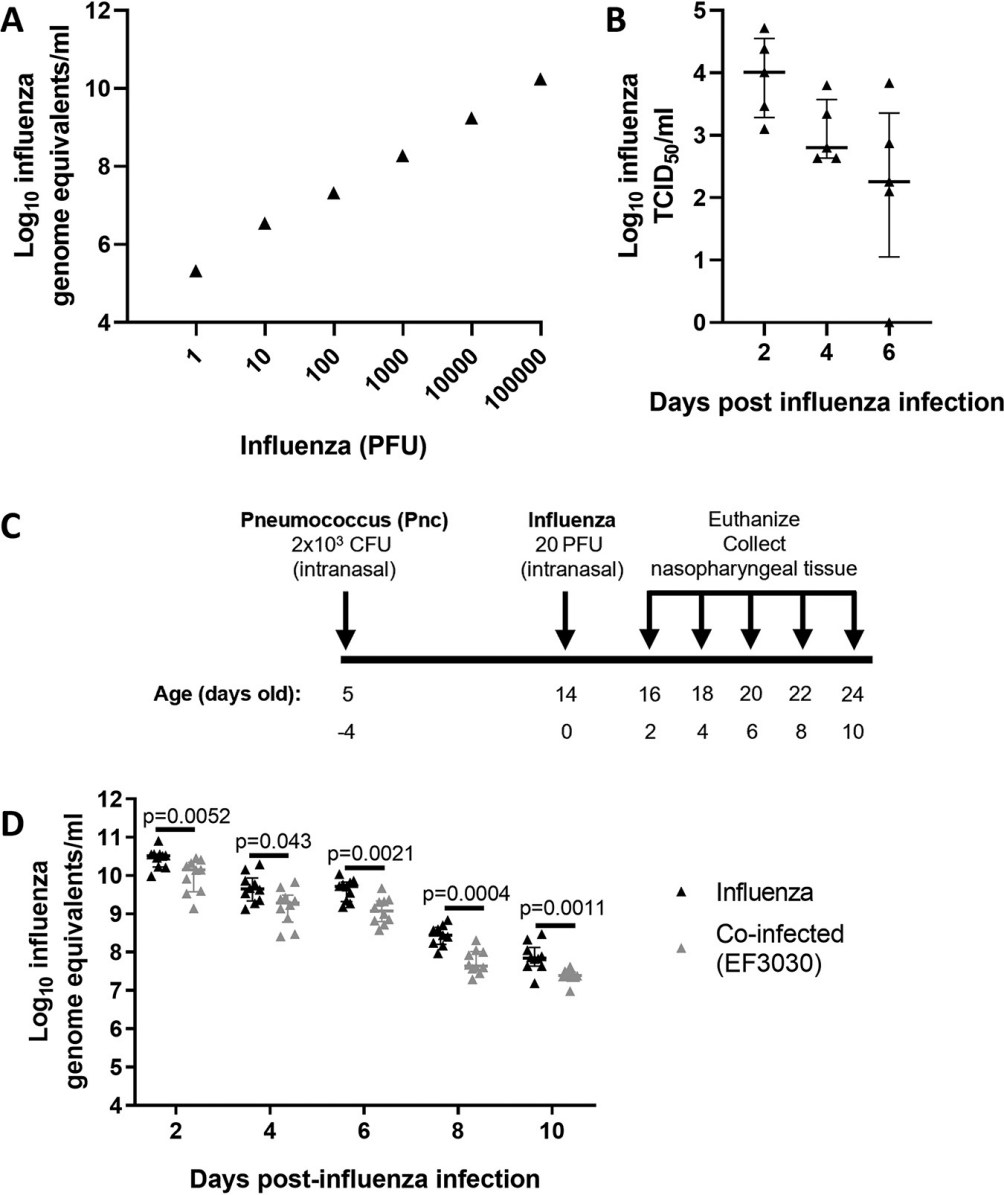
Effect of coinfection on influenza. (A) Correlation of the influenza qRT-PCR assay with infectious virus. (B) Detection of infectious influenza virus from mice infected with 20 PFU at 14 days of age. (C) Schematic of the pneumococcal-influenza viral coinfection model. PBS was administered as a vehicle control. (D) Influenza virus loads over time in mice coinfected with pneumococci (EF3030), compared with those in mice given influenza virus alone (*n* = 9 to 10 mice per group). Data are presented as medians ± IQR and were analyzed by the Mann-Whitney test. Only *P* values of <0.05 are shown. In the qRT-PCR assay, 20 PFU of influenza virus is equivalent to 6.85 log_10_ genome equivalents/ml.

Previously, we had identified that infant mice colonized with pneumococci had reduced influenza loads in the nasopharynx (13). We therefore hypothesized that pneumococcal colonization accelerates the clearance of influenza from the nasopharynx in a manner similar to its effect on PVM. We first established a qRT-PCR that would allow quantification of influenza loads. As with PVM, the assay for influenza overestimates viral loads, but loads still correlate with infectious virus (Fig. 6A) (*P* = 0.001, *r* = 1.00, Spearman’s correlation). In line with the ability of influenza virus to replicate in the upper respiratory tract (47), we confirmed that infectious virus could be recovered from mice by determining the TCID_50_ (Fig. 6B), which correlated with our qRT-PCR assay results (*r* = 0.86, *P* < 0.0001, Spearman's correlation).

To ascertain the effect of pneumococci on influenza, we administered 20 PFU of influenza virus to 14-day-old mice (equivalent to 6.85 log_10_ influenza genome equivalents/ml) 9 days after they had received pneumococci and measured influenza loads in the nasopharynx over time (Fig. 6C). Compared with mice given influenza only, mice colonized with pneumococci had reduced influenza loads at all tested time points (Fig. 6D). There were 0.37, 0.37, 0.64, 0.81, and 0.46 log_10_ reductions in influenza loads in EF3030-colonized mice at 2, 4, 6, 8, and 10 days after influenza infection (*P* < 0.05 for all time points, Mann-Whitney test), respectively. Unlike with PVM, influenza loads in coinfected mice were never comparable to those in mice given influenza alone.

## DISCUSSION

In this study, we developed a novel model of pneumococcal-RSV coinfection using PVM, the murine equivalent of RSV. Our results demonstrate the complexity of bacterial-viral interactions, identifying both synergistic and antagonistic effects during coinfection. Our mouse model employs a natural cognate virus/host approach, thereby requiring lower doses and allowing robust viral replication more reflective of a natural upper respiratory tract infection (34–36). Additionally, we use an order of infection that we hypothesize is more relevant to high-risk settings (8).

Mice that were colonized with pneumococci had inflammatory cells in their nasal tissue (Fig. 2C). This is consistent with previous studies in mice (48, 49). Pneumococcal infection of air-liquid interface cultures increases the production of inflammatory cytokines IL-1β and TNF (21). Studies involving restimulation of human nasal cells found that those derived from participants colonized with pneumococci had elevated levels of CCL3, IL-6, and TNF (50). Increased production of these chemokines and cytokines in response to pneumococcal colonization was also observed in our infant mouse model (Fig. 3). We also observed that PVM infection was associated with olfactory sensory neuronal necrosis (Fig. 2D), consistent with a mouse model of RSV which showed a tropism of RSV for nasal olfactory sensory neurons and induction of necrosis along the airway epithelium (51, 52), as well as evidence of neurological sequelae associated with RSV infection in humans (53–55). Additionally, we found increased production of CCL2 and CCL5 in the nasopharynx in response to PVM infection (Fig. 3D and G). To our knowledge, there are no studies investigating the immune response to PVM in the upper respiratory tract. However, chemokine profiles from human nasal wash samples (56, 57) and mouse lungs infected with RSV (58, 59) also show higher levels of CCL2 and CCL5.

Similar to observations in nasopharyngeal samples from RSV patients (42, 43), PVM infection increased pneumococcal nasopharyngeal density in our model (Fig. 4A and B). Elevated pneumococcal nasopharyngeal density is a risk factor for pneumonia (60, 61). Changes in the proinflammatory response in the upper respiratory tract induced by influenza infection results in the upregulation of pneumococcal receptors and increased nutrient availability, leading to enhanced bacterial adherence and growth, resulting in increased pneumococcal density (62–64). In our study, we also found that pneumococcal-PVM coinfection enhances the inflammatory response, supported by both histopathological analysis (Fig. 2) and increases in levels of proinflammatory cytokine and chemokines (Fig. 3). Therefore, although the underlying mechanism governing the PVM-mediated increase in pneumococcal density observed in our study is unclear, it is likely that the proinflammatory response to PVM infection plays a role in this process and future studies will aim to test this hypothesis.

We found that PVM infection increased pneumococcal egress (Fig. 4C and D), a critical phase of transmission (11), which begins as the virus approaches peak loads (Fig. 1B). The increase in pneumococcal shedding occurred with both strains but was more pronounced for BCH19, in both duration and magnitude. In the absence of viral infection, BCH19 shedding was ∼1 log_10_ lower than EF3030 shedding (Fig. 4C and D). It is plausible that PVM infection has a more pronounced effect on pneumococcal strains that are usually shed in lower numbers in the absence of virus.

Influenza enhances pneumococcal transmission from colonized hosts (index) to contacts in animal models (13, 15) and household contact studies (16). In contrast, a cotton rat model of pneumococcal-RSV coinfection did not find evidence that RSV increased pneumococcal transmission (65). However, the contacts in the cotton rat model were not infected with RSV, which at least in the influenza mouse model is important for facilitating pneumococcal acquisition (13, 15). Although we administered PVM to both index and contact mice, we found no significant increase in the acquisition of pneumococci in the presence of virus. Overall, our data suggest that PVM infection enhances the early stages of pneumococcal transmission (egress from a colonized host) but has more limited effects on the acquisition stage.

Our study uncovered novel evidence of pneumococcus-mediated antagonism on PVM infection. Mice colonized with pneumococci had reduced PVM loads in the upper respiratory tract (Fig. 5). Examination of viral loads over time identified that this reduction was unlikely due to pneumococcus-mediated inhibition of PVM replication, as viral loads in PVM-infected and coinfected mice peaked at the same time (6 to 8 days post-PVM infection). Additionally, the reduction in PVM nasopharyngeal loads is unlikely to be explained by increased viral shedding, as these effects occurred at different time points in the model (8 and 10 days post-PVM infection, respectively). Some significant differences were also noted in the early stages of PVM replication where mice coinfected with BCH19 had lower and higher PVM loads than in mice given PVM alone at 2 and 4 days post-PVM infection, respectively. However, the lack of a trend suggests that these differences were due to chance. In contrast, during the resolution phase, the magnitude of the reduction increased over time. By 14 days post-PVM infection, PVM was no longer detectable in 47% of pneumococcus-colonized mice but was still detectable in 100% of mice infected with PVM alone at the same time point (Fig. 5G). These data demonstrate that pneumococcal colonization may create an environment capable of accelerating the clearance of PVM from the nasopharynx.

This antagonistic effect of pneumococci on PVM was conserved regardless of the pneumococcal strain or serotype tested, or even the order of infection. When we infected mice with either ethanol-killed pneumococci or the Δ*cpsB* mutant (a mutant pneumococcal strain deficient in capsule production and incapable of carriage), this antagonistic effect was abrogated (Fig. 5E). However, the antagonism occurs with PMP1124, a nonencapsulated strain that is still capable of colonization (Fig. 5F). Our data indicate that sufficient exposure to pneumococci, such as through colonization, is required for the accelerated clearance of PVM from the upper respiratory tract to occur. Although our observed antagonistic effects have important implications for coinfection, our data suggest that the presence of colonizing pneumococci leads to viral antagonism, rather than coinfection *per se*.

In our murine coinfection model, CCL3 levels were elevated following pneumococcal colonization, and this increased even further during PVM coinfection (Fig. 3E). CCL3 is a chemoattractant for neutrophils, monocytes, dendritic cells, and T cells (66, 67). This chemokine plays a role in the clearance of microbes, such as influenza virus in the lung (68), murine cytomegalovirus in the liver and spleen (69), Klebsiella pneumoniae in the lung (70), and nontypeable Haemophilus influenzae in the middle ear (71). CCL3 knockout mice have increased PVM loads in the lungs and reduced recruitment of immune cells, demonstrating that this chemokine plays an important role in the control of PVM infection (72). Future experiments will test the role of CCL3 and other effectors in pneumococcus-mediated accelerated clearance of PVM.

Adding to the complexity of these bacterial-viral interactions, we found that the timing and magnitude of accelerated PVM clearance mediated by pneumococcal colonization varied by strain. EF3030-colonized mice had a greater reduction in PVM loads at 20 days of age (11 days post-PVM infection) than mice carrying BCH19 (Fig. 5B), and this finding was also confirmed in our time course experiments (Fig. 5G). In humans, the immune response to pneumococcal colonization positively correlates with pneumococcal density, with higher loads in the nasopharynx associated with increased levels of MMP-9, IL-6 (73), and CXCL10, as well as monocyte recruitment (50). It is therefore plausible that the timing and magnitude of pneumococcus-mediated clearance of PVM is influenced by bacterial density, with high-density-colonization strains eliciting a greater immune response, leading to a stronger and earlier anti-PVM response.

The magnitudes of the synergistic and antagonistic effects observed were dependent on the pneumococcal strain. PVM infection had a greater positive effect on BCH19 than EF3030 infection (Fig. 5C and 4D). In contrast, EF3030 had a stronger and earlier negative effect on PVM than BCH19 (Fig. 5B and G). As both strains are serotype 19F, these differences cannot be attributed to the pneumococcal capsule type. One striking difference of these strains is the density to which they colonize the murine nasopharynx in the absence of coinfection (Fig. 4A), which can influence pneumococcal shedding (74) and the immune response, which may be relevant to antagonism. Future studies should focus on investigating strain-specific factors, including the role of pneumococcal density in determining the magnitude of synergistic and antagonistic outcomes of coinfection.

Lastly, we found that mice carrying pneumococci also had an antagonistic effect on influenza loads in the nasopharynx (Fig. 6D). However, the reduction in influenza loads in coinfected mice compared with controls given influenza alone occurred across all time points tested. This suggests that pneumococcal colonization may act to restrict replication of influenza rather than accelerate clearance. The inhibitory effect of pneumococci on influenza replication was also recently reported in a ferret model of pneumococcus-influenza virus coinfection (75), demonstrating the relevance of our findings beyond our mouse model. Consistent with the findings of Ortigoza et al. (76), Mueller et al. (75) showed that pneumococcal carriage reduced influenza transmission, a key step in viral pathogenesis.

Interestingly, although pneumococcal carriage could antagonize both PVM and influenza in the upper respiratory tract, these respiratory viruses were affected in different ways. That is, pneumococci accelerated the clearance of PVM late in the model but restricted replication of influenza virus at all the time points tested. The differential effects of pneumococcal carriage on these viruses suggest that the underlying mechanism governing these antagonistic effects may differ. Future studies will focus on deciphering these mechanisms.

One limitation of our study was the use of qRT-PCR for the quantification of viral loads, a method that cannot differentiate between infectious and noninfectious viral particles. Additionally, the reverse transcription step in the qRT-PCR, where more than one cDNA is synthesized per viral RNA genome, results in an overestimation of viral load. However, there was a strong positive correlation with numbers of viral genome copies detected by qRT-PCR and titers of infectious virus determined by TCID_50_ in the same samples, supporting the application of qRT-PCR to measure differences in viral loads between control and coinfected groups.

Bacterial-viral interactions have important implications for vaccination strategies. For example, coadministration of whole inactivated influenza and pneumococcal vaccines boosts protection in mice subsequently challenged with a lethal dose of influenza virus (77). A randomized control trial by Harris and colleagues identified that modulating the gut microbiome using the narrow-spectrum antibiotic vancomycin improved rotavirus vaccine immunogenicity (78). More recently, a clinical trial testing Mycobacterium bovis BCG, a vaccine for tuberculosis, had off-target protective effects against viral respiratory infections (79). Therefore, the pneumococcus-mediated viral antagonism observed in our study may have implications for bacterial and viral vaccines. For example, development of a pneumococcal vaccine that results in viral antagonism may also reduce viral disease. In contrast, if vaccinees carry pneumococci at the time of vaccination, the protection conferred by live attenuated viral vaccines may be reduced. Interestingly, a recent human challenge study found that the immune response to live attenuated influenza vaccine was impaired in adults colonized with pneumococci (80), with some evidence for reduced viral loads (as inferred from qPCR threshold cycle [*C_T_*] values) in colonized individuals.

Here, we have developed and tested a novel model of pneumococcal-RSV coinfection that is the first to show both synergistic and antagonistic interplay between these two pathogens. Taken together, our study demonstrates that the dynamics of pneumococcal-viral coinfection are more complex than initially anticipated. This study provides the basis for exploring the mechanism underpinning these observations and the implications of microbial interactions for bacterial and viral vaccination strategies.

## MATERIALS AND METHODS

### Bacterial and viral strains.

Four pneumococcal strains were used in this study: EF3030 (serotype 19F, multilocus sequence type 43 [ST43]) (81), BCH19 (serotype 19F, ST81), 0603 (serotype 6B, ST1536) (82), and PMP1124, a nonencapsulated (NT3b lineage) strain (83). The Δ*cpsB* mutant was constructed in a EF3030 background using genomic DNA from a previously constructed Δ*cpsB* mutant kindly provided by Alistair Standish. A vial of EF3030 culture (optical density [OD], 0.1) was incubated with 500 μl CTM (1% [wt/vol] Casamino Acids, 0.5% [wt/vol] tryptone, 0.5% [wt/vol] NaCl, 1% [wt/vol] yeast extract, 16 μM K_2_HPO_4_, 0.2% [wt/vol] glucose, 150 μg/ml glutamine) and 55 ng CSP-1 for 10 min at 37°C with 5% CO_2_ prior to the addition of ∼50 ng genomic DNA. The vial was then incubated at 32°C for 30 min and then at 37°C with 5% CO_2_ for 4 h. Transformants were isolated on horse blood agar (HBA) supplemented with 0.2 μg/ml erythromycin. Deletion of the *cpsB* gene was validated by whole-genome sequencing. To prepare infectious stocks, strains were grown in THY broth (3% [wt/vol] Todd-Hewitt broth, 0.5% [wt/vol] yeast extract) statically at 37°C with 5% CO_2_ to an OD at 600 nm (OD_600_) of ∼0.4. Glycerol was added to a final concentration of 8% (vol/vol), and cultures were aliquoted and stored at −80°C until required. Ethanol-killed pneumococci were prepared by harvesting bacteria, resuspending them in an equal volume of absolute ethanol, and incubating the mixture at room temperature for 15 min. Following centrifugation to pellet the bacteria, the ethanol was removed and the pellets were allowed to air dry prior to their resuspension in an equal volume of phosphate-buffered saline (PBS). Preparations were plated on HBA to ensure that no viable pneumococci remained.

PVM strain J3666 was used in this study. Infectious stocks were prepared by purification of PVM virions from mouse lung homogenates as described previously (84). Inactivation of PVM was achieved by exposure of 10 PFU in a 3-μl preparation to UV light (30 W, G30T8) for 20 min. qRT-PCR of the preparation was performed to ensure that PVM detection was not affected by UV treatment and was found to have no impact on the ability of the assay to detect PVM. Influenza strain A/Udorn/307/72 (H3N2) was used for this study, and stocks were prepared as described previously (85).

### Infant mouse model.

All mouse experiments were conducted under approval of the Murdoch Children’s Research Institute (MCRI) Animal Ethics Committee (protocols A832, A857, and A898) in accordance with the Australian code for the care and use of animals for scientific purposes (90). C57BL/6 mice were imported from the Walter and Eliza Hall Institute and housed at the animal facility at MCRI with unlimited access to food and water. At 5 days of age, pups were given 2 × 10^3^ CFU of pneumococci in 3 μl PBS intranasally without anesthesia. At 9 days of age, pups were administered 10 PFU of PVM in 3 μl PBS intranasally without anesthesia. For studies involving influenza, pups were administered 20 PFU of influenza in 3 μl PBS intranasally without anesthesia at 14 days of age. Any control groups not receiving both microbes (e.g., mock- or mono-infection groups) were administered PBS as a vehicle control. Pups in this model did not exhibit any overt clinical signs of illness and were housed with their dam for the duration of the experiment. Unless otherwise stated, mice were euthanized at 20 days by cervical dislocation following anesthesia. Nasopharyngeal tissue was harvested, placed in 1.5 ml RPMI 1640 medium (Sigma-Aldrich), and homogenized. For the enumeration of pneumococci, tissue homogenates were serially diluted and plated on HBA supplemented with 5 μg/ml gentamicin to select for pneumococci. To quantify viral loads, nasopharyngeal homogenates were subjected to centrifugation at 1,400 × *g* for 10 min at 4°C. The supernatants were then used for viral RNA extraction and qRT-PCR.

### Pneumococcal and viral shedding.

Shedding of pneumococci in the nasal secretions of mice was evaluated using a method adapted from previously described approaches (23, 86). Briefly, the nose of the mouse was tapped 5 times across two HBA plates supplemented with 5 μg/ml gentamicin. Using a 10-μl disposable loop dipped in PBS, the nasal secretions were spread across the plate. Plates were then incubated as described above and colonies counted to calculate the number of CFU/5 taps.

To assess shedding of PVM in mouse nasal secretions, an infant FLOQSwab (Copan) was moistened in PBS and used to swab the anterior nares of the mouse five times. The swab was then placed in 200 μl of PBS. Samples were vortexed for 5 s and stored at −80°C until required for viral RNA extraction and qRT-PCR.

### Extraction of viral RNA and qRT-PCR detection of PVM or influenza virus.

Extraction of viral RNA from the supernatants of nasopharyngeal homogenates or swab aliquots was performed using a QIAamp viral RNA mini kit (Qiagen) as per the manufacturer’s instructions, except that RNA was eluted in 30 μl of AVE buffer. Detection and quantification of viruses were conducted by qRT-PCR using the SensiFAST probe no-ROX one-step kit (Bioline). The assay for detection of PVM uses primers and a probe targeting the SH gene (forward primer, 5′-ATGACCAGCAGCCGCATTGG-3′; reverse primer, 5′-TGCTTCTACTGCTGCAGGCC-3′; probe, 6FAM-CCTAACAGCTCTTCTCCTTGCATGTGC-BHQ1, where 6FAM is 6-carboxyfluorescein). The assay for detection of influenza targets the M gene using primer and probe sequences reported previously (87) (forward primer, 5′-GGACTGCAGCGTAGACGCTT-3′; reverse primer, 5′-CATCCTGTTGTATATGAGGCCCAT-3′; probe, 6FAM-CTCAGTTATTCTGCTGGTGCACTTGCCA-BHQ1). To each 20-μl reaction mixture containing master mix, 0.4 μM each primer, and 0.1 μM probe, 4 μl of viral RNA was added and run under the following cycling conditions: 45°C for 10 min, 95°C for 2 min, and 40 cycles of 95°C for 15 s and 60°C for 30 s. Viral genome copy number was calculated using a standard curve prepared with vector DNA consisting of the SH or M gene cloned into pGEM-T Easy (Promega) and expressed as log_10_ numbers of genome equivalents/ml of eluted RNA. In the qRT-PCR assays, 10 PFU of PVM and 20 PFU of influenza virus was equivalent to 7.33 and 6.85 log_10_ genome equivalents/ml for PVM and influenza virus, respectively.

### TCID_50_.

The TCID_50_ assay for influenza was performed as described previously (88, 89) using confluent MDCK cells. For PVM, the TCID_50_ was measured using LLC-MK2 (*Macaca* kidney epithelial) cells. Briefly, 50 μl of 10-fold serial dilutions of mouse tissue homogenate supernatants was added to MDCK (4 × 10^5^) or LLC-MK2 (1 × 10^4^) cells and incubated for 1 h to allow viral attachment. Cells were washed with PBS, fresh Dulbecco’s modified Eagle’s medium (DMEM) was added (supplemented with 5% fetal calf serum [Sigma-Aldrich] for the PVM TCID_50_), and the mixtures were incubated at 37°C with 5% CO_2_ for 3 and 7 days for influenza virus and PVM, respectively. For influenza virus, wells with cytopathic effects were defined as positive, as described previously (88, 89). In contrast, as PVM recovered from mouse tissue induces very minor cytopathic effects *in vitro*, qRT-PCR was used. A positive well was defined as a reduction in the *C_T_* value 7 days postinfection of LLC-MK2 cells compared with that at day 0, indicative of an increase in viral copy number over time and therefore the presence of infectious, replicating virus in that well.

### Cytometric bead array.

Nasopharyngeal homogenates were prepared as described above, except that tissues were homogenized in 500 μl RPMI 1640, cell debris was pelleted by centrifugation (1,400 × *g* for 10 min at 4°C), and supernatants were assayed for chemokine and cytokine levels using the CBA flex set (BD Bioscience) in accordance with the manufacturer’s instructions. Data were acquired using a FACSCanto II flow cytometer and concentrations calculated by comparing results to standard curves generated for each chemokine and cytokine.

### Histopathological analysis of nasopharyngeal tissue.

Following euthanasia, mouse heads were fixed in 10% (vol/vol) neutral buffered formalin and sent to the Australian Phenomics Network Histopathology and Organ Pathology Service at The University of Melbourne for sectioning and histopathological analysis. Sections (5-μm thickness) were prepared from the heads at five rostral levels at 200 μm apart. For the assessment of inflammation and tissue damage, hematoxylin and eosin stainings were conducted on tissue sections. Samples were scored for histopathological changes. Samples were scored for exudate within the lumen of the nasal cavity, inflammation/number of inflammatory cells, and epithelial changes (e.g., loss of cilia, loss of epithelial cell layers). Scoring was conducted by the Australian Phenomics Network Histopathology and Organ Pathology Service, The University of Melbourne, and an independent veterinary pathologist. Both operators were blind to infection group.

### Statistical analysis.

All graphs were produced and statistical analyses were conducted using GraphPad Prism version 8.4.2 (GraphPad Software). Data that were normally distributed were expressed as means ± standard deviations and analyzed by an unpaired *t* test or one-way ANOVA for Tukey’s tests for pairwise comparisons. Otherwise, data were log_10_ transformed and expressed as the medians ± IQRs, and groups were compared by the Mann-Whitney test. Categorical data were analyzed by Fisher’s exact test. For all statistical analyses, an α cutoff of 0.05 was used to define a significant difference between groups. Data where correlations or associations were examined between infectious virus and qRT-PCR were analyzed by Spearman’s correlation.

To determine if the increased effects on nasal tissue damage and CCL3 levels during coinfection were synergistic, we adapted the Bliss independence model (41), which predicts the expected combined effect of multiple stimuli. The Bliss independence model is defined as follows: *f_xy_*_,_ *_P_ = f_x_ + f_y_ −* (*f_x_*)(*f_y_*), where *f_x_* and *f_y_* are the effects of stimuli *x* and *y*, respectively. The relationship was evaluated by comparing the experimentally observed effect of coinfection (*f_xy_*_,_ *_O_*) to the predicted (additive) effect (*f_xy_*_,_ *_P_*), and the Bliss independence score was calculated using the formula *f_xy_*_,_ *_O_* − *f_xy_*_,_ *_P_*. A Bliss independence score of 0, >0, or <0 indicates an independent (additive), synergistic, or antagonistic effect of coinfection, respectively.

## References

[1] Neu U, Mainou BA. 2020. Virus interactions with bacteria: partners in the infectious dance. PLoS Pathog 16:e1008234. doi:10.1371/journal.ppat.1008234. 32045465 PMC7012391

[2] Barr R, Green CA, Sande CJ, Drysdale SB. 2019. Respiratory syncytial virus: diagnosis, prevention and management. Ther Adv Infect Dis 6:2049936119865798. doi:10.1177/2049936119865798. 31384456 PMC6664627

[3] Zhou F, Yu T, Du R, Fan G, Liu Y, Liu Z, Xiang J, Wang Y, Song B, Gu X, Guan L, Wei Y, Li H, Wu X, Xu J, Tu S, Zhang Y, Chen H, Cao B. 2020. Clinical course and risk factors for mortality of adult inpatients with COVID-19 in Wuhan, China: a retrospective cohort study. Lancet 395:1054–1062. doi:10.1016/S0140-6736(20)30566-3. 32171076 PMC7270627

[4] Lee H, Ko GP. 2016. Antiviral effect of vitamin A on norovirus infection via modulation of the gut microbiome. Sci Rep 6:25835. doi:10.1038/srep25835. 27180604 PMC4867650

[5] Clark SE. 2020. Commensal bacteria in the upper respiratory tract regulate susceptibility to infection. Curr Opin Immunol 66:42–49. doi:10.1016/j.coi.2020.03.010. 32416468 PMC7665980

[6] Schubert AM, Rogers MAM, Ring C, Mogle J, Petrosino JP, Young VB, Aronoff DM, Schloss PD. 2014. Microbiome data distinguish patients with *Clostridium difficile* infection and non-*C. difficile*-associated diarrhea from healthy controls. mBio 5:e01021-14. doi:10.1128/mBio.01021-14. 24803517 PMC4010826

[7] O'Brien KL, Wolfson LJ, Watt JP, Henkle E, Deloria-Knoll M, McCall N, Lee E, Mulholland K, Levine OS, Cherian T, Hib and Pneumococcal Global Burden of Disease Study Team. 2009. Burden of disease caused by *Streptococcus pneumoniae* in children younger than 5 years: global estimates. Lancet 374:893–902. doi:10.1016/S0140-6736(09)61204-6. 19748398

[8] Adegbola RA, DeAntonio R, Hill PC, Roca A, Usuf E, Hoet B, Greenwood BM. 2014. Carriage of *Streptococcus pneumoniae* and other respiratory bacterial pathogens in low and lower-middle income countries: a systematic review and meta-analysis. PLoS One 9:e103293. doi:10.1371/journal.pone.0103293. 25084351 PMC4118866

[9] Kadioglu A, Weiser JN, Paton JC, Andrew PW. 2008. The role of *Streptococcus pneumoniae* virulence factors in host respiratory colonization and disease. Nat Rev Microbiol 6:288–301. doi:10.1038/nrmicro1871. 18340341

[10] Simell B, Auranen K, Käyhty H, Goldblatt D, Dagan R, O'Brien KL, Pneumococcal Carriage Group. 2012. The fundamental link between pneumococcal carriage and disease. Expert Rev Vaccines 11:841–855. doi:10.1586/erv.12.53. 22913260

[11] Weiser JN, Ferreira DM, Paton JC. 2018. *Streptococcus pneumoniae*: transmission, colonization and invasion. Nat Rev Microbiol 16:355–367. doi:10.1038/s41579-018-0001-8. 29599457 PMC5949087

[12] Siemens N, Oehmcke-Hecht S, Mettenleiter TC, Kreikemeyer B, Valentin-Weigand P, Hammerschmidt S. 2017. Port d’entrée for respiratory infections—does the influenza A virus pave the way for bacteria? Front Microbiol 8:2602–2617. doi:10.3389/fmicb.2017.02602. 29312268 PMC5742597

[13] Diavatopoulos DA, Short KR, Price JT, Wilksch JJ, Brown LE, Briles DE, Strugnell RA, Wijburg OL. 2010. Influenza A virus facilitates *Streptococcus pneumoniae* transmission and disease. FASEB J 24:1789–1798. doi:10.1096/fj.09-146779. 20097876

[14] Short KR, Reading PC, Wang N, Diavatopoulos DA, Wijburg OL. 2012. Increased nasopharyngeal bacterial titers and local inflammation facilitate transmission of *Streptococcus pneumoniae*. mBio 3:e00255-12. doi:10.1128/mBio.00255-12. 23015738 PMC3518912

[15] McCullers JA, McAuley JL, Browall S, Iverson AR, Boyd KL, Henriques Normark B. 2010. Influenza enhances susceptibility to natural acquisition of and disease due to *Streptococcus pneumoniae* in ferrets. J Infect Dis 202:1287–1295. doi:10.1086/656333. 20822454 PMC3249639

[16] Ujiie M, Izumi S, Takeshit N, Takasaki J, Mizuno Y, Kato Y, Kanagaw S, Kudo K. 2010. Household transmission of pneumococcal pneumonia associated with pandemic influenza (H1N1) 2009. Nihon Kokyuki Gakkai Zasshi 48:322–327. (In Japanese.) 20432976

[17] Brealey JC, Chappell KJ, Galbraith S, Fantino E, Gaydon J, Tozer S, Young PR, Holt PG, Sly PD. 2018. *Streptococcus pneumoniae* colonization of the nasopharynx is associated with increased severity during respiratory syncytial virus infection in young children. Respirology 23:220–227. doi:10.1111/resp.13179. 28913912 PMC7169064

[18] Resch B, Gusenleitner W, Mueller WD. 2007. Risk of concurrent bacterial infection in preterm infants hospitalized due to respiratory syncytial virus infection. Acta Paediatr 96:495–498. doi:10.1111/j.1651-2227.2007.00226.x. 17326757

[19] Fathima P, Blyth CC, Lehmann D, Lim FJ, Abdalla T, de Klerk N, Moore HC. 2018. The impact of pneumococcal vaccination on bacterial and viral pneumonia in Western Australian children: record linkage cohort study of 469589 births, 1996–2012. Clin Infect Dis 66:1075–1085. doi:10.1093/cid/cix923. 29069315

[20] Weinberger DM, Klugman KP, Steiner CA, Simonsen L, Viboud C. 2015. Association between respiratory syncytial virus activity and pneumococcal disease in infants: a time series analysis of US hospitalization data. PLoS Med 12:e1001776. doi:10.1371/journal.pmed.1001776. 25562317 PMC4285401

[21] Smith CM, Sandrini S, Datta S, Freestone P, Shafeeq S, Radhakrishnan P, Williams G, Glenn SM, Kuipers OP, Hirst RA, Easton AJ, Andrew PW, O'Callaghan C. 2014. Respiratory syncytial virus increases the virulence of *Streptococcus pneumoniae* by binding to penicillin binding protein 1a. A new paradigm in respiratory infection. Am J Respir Crit Care Med 190:196–207. doi:10.1164/rccm.201311-2110OC. 24941423 PMC4226051

[22] Simon AK, Hollander GA, McMichael A. 2015. Evolution of the immune system in humans from infancy to old age. Proc Biol Sci 282:20143085. doi:10.1098/rspb.2014.3085. 26702035 PMC4707740

[23] Richard AL, Siegel SJ, Erikson J, Weiser JN. 2014. TLR2 signaling decreases transmission of *Streptococcus pneumoniae* by limiting bacterial shedding in an infant mouse influenza A co-infection model. PLoS Pathog 10:e1004339. doi:10.1371/journal.ppat.1004339. 25166617 PMC4148449

[24] Siegrist CA. 2001. Neonatal and early life vaccinology. Vaccine 19:3331–3346. doi:10.1016/S0264-410X(01)00028-7. 11348697

[25] Brealey JC, Young PR, Sloots TP, Ware RS, Lambert SB, Sly PD, Grimwood K, Chappell KJ. 2020. Bacterial colonization dynamics associated with respiratory syncytial virus during early childhood. Pediatr Pulmonol 55:1237–1245. doi:10.1002/ppul.24715. 32176838

[26] Dakhama A, Park J-W, Taube C, Joetham A, Balhorn A, Miyahara N, Takeda K, Gelfand EW. 2005. The enhancement or prevention of airway hyperresponsiveness during reinfection with respiratory syncytial virus is critically dependent on the age at first infection and IL-13 production. J Immunol 175:1876–1883. doi:10.4049/jimmunol.175.3.1876. 16034131

[27] Taylor G, Stott EJ, Hughes M, Collins AP. 1984. Respiratory syncytial virus infection in mice. Infect Immun 43:649–655. doi:10.1128/iai.43.2.649-655.1984. 6693171 PMC264349

[28] Ruckwardt TJ, Malloy AMW, Gostick E, Price DA, Dash P, McClaren JL, Thomas PG, Graham BS. 2011. Neonatal CD8 T-cell hierarchy is distinct from adults and is influenced by intrinsic T cell properties in respiratory syncytial virus infected mice. PLoS Pathog 7:e1002377. doi:10.1371/journal.ppat.1002377. 22144888 PMC3228797

[29] Stokes KL, Chi MH, Sakamoto K, Newcomb DC, Currier MG, Huckabee MM, Lee S, Goleniewska K, Pretto C, Williams JV, Hotard A, Sherrill TP, Peebles RS, Moore ML. 2011. Differential pathogenesis of respiratory syncytial virus clinical isolates in BALB/c mice. J Virol 85:5782–5793. doi:10.1128/JVI.01693-10. 21471228 PMC3126300

[30] Hament J-M, Aerts PC, Fleer A, van Dijk H, Harmsen T, Kimpen JLL, Wolfs TFW. 2005. Direct binding of respiratory syncytial virus to pneumococci: a phenomenon that enhances both pneumococcal adherence to human epithelial cells and pneumococcal invasiveness in a murine model. Pediatr Res 58:1198–1203. doi:10.1203/01.pdr.0000188699.55279.1b. 16306193

[31] Hall CB, Douglas RG, Schnabel KC, Geiman JM. 1981. Infectivity of respiratory syncytial virus by various routes of inoculation. Infect Immun 33:779–783. doi:10.1128/iai.33.3.779-783.1981. 7287181 PMC350778

[32] Kravetz HM, Knight V, Chanock RM, Morris JA, Johnson KM, Rifkind D, Utz JP. 1961. Respiratory syncytial virus. III. Production of illness and clinical observations in adult volunteers. JAMA 176:657–663. 13754148

[33] Yezli S, Otter JA. 2011. Minimum infective dose of the major human respiratory and enteric viruses transmitted through food and the environment. Food Environ Virol 3:1–30. doi:10.1007/s12560-011-9056-7. PMC7090536 35255645

[34] Bem RA, Domachowske JB, Rosenberg HF. 2011. Animal models of human respiratory syncytial virus disease. Am J Physiol Lung Cell Mol Physiol 301:L148–L156. doi:10.1152/ajplung.00065.2011. 21571908 PMC3154630

[35] Sacco RE, Durbin RK, Durbin JE. 2015. Animal models of respiratory syncytial virus infection and disease. Curr Opin Virol 13:117–122. doi:10.1016/j.coviro.2015.06.003. 26176495 PMC4699663

[36] Rosenberg HF, Domachowske JB. 2008. Pneumonia virus of mice: severe respiratory infection in a natural host. Immunol Lett 118:6–12. doi:10.1016/j.imlet.2008.03.013. 18471897 PMC2494858

[37] Lynch JP, Werder RB, Simpson J, Loh Z, Zhang V, Haque A, Spann K, Sly PD, Mazzone SB, Upham JW, Phipps S. 2016. Aeroallergen-induced IL-33 predisposes to respiratory virus-induced asthma by dampening antiviral immunity. J Allergy Clin Immunol 138:1326–1337. doi:10.1016/j.jaci.2016.02.039. 27236500

[38] Dyer KD, Schellens IM, Bonville CA, Martin BV, Domachowske JB, Rosenberg HF. 2007. Efficient replication of pneumonia virus of mice (PVM) in a mouse macrophage cell line. Virol J 4:48. doi:10.1186/1743-422X-4-48. 17547763 PMC1891281

[39] Arikkatt J, Ullah MA, Short KR, Zhang V, Gan WJ, Loh Z, Werder RB, Simpson J, Sly PD, Mazzone SB, Spann KM, Ferreira MA, Upham JW, Sukkar MB, Phipps S. 2017. RAGE deficiency predisposes mice to virus-induced paucigranulocytic asthma. Elife 6:e21199. doi:10.7554/eLife.21199. 28099113 PMC5243115

[40] Werder RB, Lynch JP, Simpson JC, Zhang V, Hodge NH, Poh M. 2018. PGD2/DP2 receptor activation promotes severe viral bronchiolitis by suppressing IFN-production. Sci Transl Med 10:52. doi:10.1126/scitranslmed.aao0052. 29743346

[41] Bliss CI. 1939. The toxicity of poisons applied jointly. Ann Appl Biol 26:585–615. doi:10.1111/j.1744-7348.1939.tb06990.x.

[42] Chappell K, Brealey J, Mackay I, Bletchly C, Hugenholtz P, Sloots T. 2013. Respiratory syncytial virus infection is associated with increased bacterial load in the upper respiratory tract in young children. J Med Microbiol Diagnosis S1:005. doi:10.4172/2161-0703.s1-005.

[43] Wolter N, Tempia S, Cohen C, Madhi SA, Venter M, Moyes J, Walaza S, Malope-Kgokong B, Groome M, Du Plessis M, Magomani V, Pretorius M, Hellferscee O, Dawood H, Kahn K, Variava E, Klugman KP, von Gottberg A. 2014. High nasopharyngeal pneumococcal density, increased by viral coinfection, is associated with invasive pneumococcal pneumonia. J Infect Dis 210:1649–1657. doi:10.1093/infdis/jiu326. 24907383

[44] Morona JK, Morona R, Miller DC, Paton JC. 2002. *Streptococcus pneumoniae* capsule biosynthesis protein CpsB is a novel manganese-dependent phosphotyrosine-protein phosphatase. J Bacteriol 184:577–583. doi:10.1128/JB.184.2.577-583.2002. 11751838 PMC139577

[45] Morona JK, Morona R, Paton JC. 2006. Attachment of capsular polysaccharide to the cell wall of *Streptococcus pneumoniae* type 2 is required for invasive disease. Proc Natl Acad Sci USA 103:8505–8510. doi:10.1073/pnas.0602148103. 16707578 PMC1482522

[46] Morona JK, Miller DC, Morona R, Paton JC. 2004. The effect that mutations in the conserved capsular polysaccharide biosynthesis genes *cpsA, cpsB,* and *cpsD* have on virulence of *Streptococcus pneumoniae*. J Infect Dis 189:1905–1913. doi:10.1086/383352. 15122528

[47] Hatta M, Hatta Y, Kim JH, Watanabe S, Shinya K, Nguyen T, Lien PS, Le QM, Kawaoka Y. 2007. Growth of H5N1 influenza A viruses in the upper respiratory tracts of mice. PLoS Pathog 3:e133. doi:10.1371/journal.ppat.0030133. PMC2000968 17922570

[48] Van Rossum AMC, Lysenko ES, Weiser JN. 2005. Host and bacterial factors contributing to the clearance of colonization by *Streptococcus pneumoniae* in a murine model. Infect Immun 73:7718–7726. doi:10.1128/IAI.73.11.7718-7726.2005. 16239576 PMC1273875

[49] Zhang Z, Clarke TB, Weiser JN. 2009. Cellular effectors mediating Th17-dependent clearance of pneumococcal colonization in mice. J Clin Invest 119:1899–1909. doi:10.1172/JCI36731. 19509469 PMC2701860

[50] Jochems SP, Marcon F, Carniel BF, Holloway M, Mitsi E, Smith E, Gritzfeld JF, Solórzano C, Reiné J, Pojar S, Nikolaou E, German EL, Hyder-Wright A, Hill H, Hales C, de Steenhuijsen Piters WAA, Bogaert D, Adler H, Zaidi S, Connor V, Gordon SB, Rylance J, Nakaya HI, Ferreira DM. 2018. Inflammation induced by influenza virus impairs human innate immune control of pneumococcus. Nat Immunol 19:1299–1308. doi:10.1038/s41590-018-0231-y. 30374129 PMC6241853

[51] Bryche B, Frétaud M, Saint-Albin Deliot A, Galloux M, Sedano L, Langevin C, Descamps D, Rameix-Welti M-A, Eléouët J-F, Le Goffic R, Meunier N. 2020. Respiratory syncytial virus tropism for olfactory sensory neurons in mice. J Neurochem 155:137–153. doi:10.1111/jnc.14936. 31811775

[52] Easton AJ, Domachowske JB, Rosenberg HF. 2004. Animal pneumoviruses: molecular genetics and pathogenesis. Clin Microbiol Rev 17:390–412. doi:10.1128/CMR.17.2.390-412.2004. 15084507 PMC387412

[53] Morichi S, Morishita N, Ishida Y, Oana S, Yamanaka G, Kashiwagi Y, Kawashima H. 2017. Examination of neurological prognostic markers in patients with respiratory syncytial virus-associated encephalopathy. Int J Neurosci 127:44–50. doi:10.3109/00207454.2016.1138951. 26732732

[54] Kawashima H, Ioi H, Ushio M, Yamanaka G, Matsumoto S, Nakayama T. 2009. Cerebrospinal fluid analysis in children with seizures from respiratory syncytial virus infection. Scand J Infect Dis 41:228–231. doi:10.1080/00365540802669543. 19117245

[55] Ng YT, Cox C, Atkins J, Butler IJ. 2001. Encephalopathy associated with respiratory syncytial virus bronchiolitis. J Child Neurol 16:105–108. doi:10.1177/088307380101600207. 11292214

[56] Tabarani CM, Bonville CA, Suryadevara M, Branigan P, Wang D, Huang D, Rosenberg HF, Domachowske JB. 2013. Novel inflammatory markers, clinical risk factors and virus type associated with severe respiratory syncytial virus infection. Pediatr Infect Dis J 32:e437–e442. doi:10.1097/INF.0b013e3182a14407. 23804121 PMC3883981

[57] Noah TL, Ivins SS, Murphy P, Kazachkova I, Moats-Staats B, Henderson FW. 2002. Chemokines and inflammation in the nasal passages of infants with respiratory syncytial virus bronchiolitis. Clin Immunol 104:86–95. doi:10.1006/clim.2002.5248. 12139952

[58] Goritzka M, Makris S, Kausar F, Durant LR, Pereira C, Kumagai Y, Culley FJ, Mack M, Akira S, Johansson C. 2015. Alveolar macrophage-derived type I interferons orchestrate innate immunity to RSV through recruitment of antiviral monocytes. J Exp Med 212:699–714. doi:10.1084/jem.20140825. 25897172 PMC4419339

[59] Miller AL, Bowlin TL, Lukacs NW. 2004. Respiratory syncytial virus-induced chemokine production: linking viral replication to chemokine production *in vitro* and *in vivo*. J Infect Dis 189:1419–1430. doi:10.1086/382958. 15073679

[60] Alpkvist H, Athlin S, Nauclér P, Herrmann B, Abdeldaim G, Slotved H-C, Hedlund J, Strålin K. 2015. Clinical and microbiological factors associated with high nasopharyngeal pneumococcal density in patients with pneumococcal pneumonia. PLoS One 10:e0140112. doi:10.1371/journal.pone.0140112. 26466142 PMC4605601

[61] Carr OJJ, Vilivong K, Bounvilay L, Dunne EM, Lai JYR, Chan J, Vongsakid M, Chanthongthip A, Siladeth C, Ortika B, Nguyen C, Mayxay M, Newton PN, Mulholland K, Do LAH, Dubot-Pérès A, Satzke C, Dance DAB, Russell FM. 11 May 2021. Nasopharyngeal pneumococcal colonization density is associated with severe pneumonia in young children in the Lao PDR. J Infect Dis doi:10.1093/infdis/jiab239. PMC8974848 33974708

[62] Nakamura S, Davis KM, Weiser JN. 2011. Synergistic stimulation of type I interferons during influenza virus coinfection promotes *Streptococcus pneumoniae* colonization in mice. J Clin Invest 121:3657–3665. doi:10.1172/JCI57762. 21841308 PMC3163966

[63] Siegel SJ, Roche AM, Weiser JN. 2014. Influenza promotes pneumococcal growth during coinfection by providing host sialylated substrates as a nutrient source. Cell Host Microbe 16:55–67. doi:10.1016/j.chom.2014.06.005. 25011108 PMC4096718

[64] Avadhanula V, Rodriguez CA, Devincenzo JP, Wang Y, Webby RJ, Ulett GC, Adderson EE. 2006. Respiratory viruses augment the adhesion of bacterial pathogens to respiratory epithelium in a viral species- and cell type-dependent manner. J Virol 80:1629–1636. doi:10.1128/JVI.80.4.1629-1636.2006. 16439519 PMC1367158

[65] Nguyen DT, Louwen R, Elberse K, van Amerongen G, Yüksel S, Luijendijk A, Osterhaus ADME, Duprex WP, de Swart RL. 2015. *Streptococcus pneumoniae* enhances human respiratory syncytial virus infection *in vitro* and *in vivo*. PLoS One 10:e0127098. doi:10.1371/journal.pone.0127098. 25970287 PMC4430531

[66] Menten P, Wuyts A, Van Damme J. 2002. Macrophage inflammatory protein-1. Cytokine Growth Factor Rev 13:455–481. doi:10.1016/S1359-6101(02)00045-X. 12401480

[67] Nuriev R, Johansson C. 2019. Chemokine regulation of inflammation during respiratory syncytial virus infection. F1000Res 8:1837. doi:10.12688/f1000research.20061.1. PMC6823903 31723414

[68] Cook DN, Beck MA, Coffman TM, Kirby SL, Sheridan JF, Pragnell IB, Smithies O. 1995. Requirement of MIP-1α for an inflammatory response to viral infection. Science 269:1583–1585. doi:10.1126/science.7667639. 7667639

[69] Salazar-Mather TP, Hamilton TA, Biron CA. 2000. A chemokine-to-cytokine-to-chemokine cascade critical in antiviral defense. J Clin Invest 105:985–993. doi:10.1172/JCI9232. 10749577 PMC377490

[70] Lindell DM, Standiford TJ, Mancuso P, Leshen ZJ, Huffnagle GB. 2001. Macrophage inflammatory protein 1α/CCL3 is required for clearance of an acute *Klebsiella pneumoniae* pulmonary infection. Infect Immun 69:6364–6369. doi:10.1128/IAI.69.10.6364-6369.2001. 11553580 PMC98771

[71] Deniffel D, Nuyen B, Pak K, Suzukawa K, Hung J, Kurabi A, Wasserman SI, Ryan AF. 2017. Otitis media and nasopharyngeal colonization in *ccl3*^–/–^ mice. Infect Immun 85:e00148-17. doi:10.1128/IAI.00148-17. 28847849 PMC5649018

[72] Domachowske JB, Bonville CA, Gao J-L, Murphy PM, Easton AJ, Rosenberg HF. 2000. The chemokine macrophage-inflammatory protein-1α and its receptor CCR1 control pulmonary inflammation and antiviral host defense in Paramyxovirus infection. J Immunol 165:2677–2682. doi:10.4049/jimmunol.165.5.2677. 10946298

[73] Vissers M, Ahout IM, van den Kieboom CH, van der Gaast-de Jongh CE, Groh L, Cremers AJ, de Groot R, de Jonge MI, Ferwerda G. 2016. High pneumococcal density correlates with more mucosal inflammation and reduced respiratory syncytial virus disease severity in infants. BMC Infect Dis 16:129. doi:10.1186/s12879-016-1454-x. 26983753 PMC4794819

[74] Zafar MA, Kono M, Wang Y, Zangari T, Weiser JN. 2016. Infant mouse model for the study of shedding and transmission during *Streptococcus pneumoniae* monoinfection. Infect Immun 84:2714–2722. doi:10.1128/IAI.00416-16. 27400721 PMC4995895

[75] Mueller KB, Le Sage V, French AJ, Jones JE, Padovani GH, Avery AJ, Myerburg MM, Schultz-Cherry S, Rosch JW, Hiller NL, Lakdawala SS. 2020. Coinfection of *Streptococcus pneumoniae* reduces airborne transmission of influenza virus. bioRxiv doi:10.1101/2020.11.10.376442.

[76] Ortigoza MB, Blaser SB, Zafar MA, Hammond AJ, Weiser JN. 2018. An infant mouse model of influenza virus transmission demonstrates the role of virus-specific shedding, humoral immunity, and sialidase expression by colonizing *Streptococcus pneumoniae*. mBio 9:e02359-18. doi:10.1128/mBio.02359-18. PMC6299224 30563897

[77] David SC, Norton T, Tyllis T, Wilson JJ, Singleton EV, Laan Z, Davies J, Hirst TR, Comerford I, McColl SR, Paton JC, Alsharifi M. 2019. Direct interaction of whole-inactivated influenza A and pneumococcal vaccines enhances influenza-specific immunity. Nat Microbiol 4:1316–1327. doi:10.1038/s41564-019-0443-4. 31110357

[78] Harris VC, Haak BW, Handley SA, Jiang B, Velasquez DE, Hykes BL, Droit L, Berbers GAM, Kemper EM, van Leeuwen EMM, Boele van Hensbroek M, Wiersinga WJ. 2018. Effect of antibiotic-mediated microbiome modulation on rotavirus vaccine immunogenicity: a human, randomized-control proof-of-concept trial. Cell Host Microbe 24:197–207.e4. doi:10.1016/j.chom.2018.07.005. 30092197 PMC11514417

[79] Giamarellos-Bourboulis EJ, Tsilika M, Moorlag S, Antonakos N, Kotsaki A, Domínguez-Andrés J, Kyriazopoulou E, Gkavogianni T, Adami M-E, Damoraki G, Koufargyris P, Karageorgos A, Bolanou A, Koenen H, van Crevel R, Droggiti D-I, Renieris G, Papadopoulos A, Netea MG. 2020. Activate: randomized clinical trial of BCG vaccination against infection in the elderly. Cell 183:315–323.e9. doi:10.1016/j.cell.2020.08.051. 32941801 PMC7462457

[80] Carniel BF, Marcon F, Rylance J, Zaidi S, Reine J, Negera E, Nikolaou E, Pojar S, Solórzano C, Collins AM, Connor V, Bogaert D, Gordon SB, Nakaya HI, Ferreira DM, Jochems SP, Mitsi E. 2020. Pneumococcal colonization impairs nasal and lung mucosal immune responses to live attenuated influenza vaccination in adults. JCI Insight 6:e141088. doi:10.1101/2020.02.24.20025098. PMC7934923 33497364

[81] Andersson B, Dahmén J, Frejd T, Leffler H, Magnusson G, Noori G, Edén CS. 1983. Identification of an active disaccharide unit of a glycoconjugate receptor for pneumococci attaching to human pharyngeal epithelial cells. J Exp Med 158:559–570. doi:10.1084/jem.158.2.559. 6886624 PMC2187347

[82] Malley R, Lipsitch M, Stack A, Saladino R, Fleisher G, Pelton S, Thompson C, Briles D, Anderson P. 2001. Intranasal immunization with killed unencapsulated whole cells prevents colonization and invasive disease by capsulated pneumococci. Infect Immun 69:4870–4873. doi:10.1128/IAI.69.8.4870-4873.2001. 11447162 PMC98576

[83] Satzke C, Dunne EM, Porter BD, Klugman KP, Mulholland EK, PneuCarriage project group. 2015. The PneuCarriage Project: a multi-centre comparative study to identify the best serotyping methods for examining pneumococcal carriage in vaccine evaluation studies. PLoS Med 12:e1001903. doi:10.1371/journal.pmed.1001903. 26575033 PMC4648509

[84] Garvey TL, Dyer KD, Ellis JA, Bonville CA, Foster B, Prussin C, Easton AJ, Domachowske JB, Rosenberg HF. 2005. Inflammatory responses to pneumovirus infection in IFN-αβR gene-deleted mice. J Immunol 175:4735–4744. doi:10.4049/jimmunol.175.7.4735. 16177121

[85] Short KR, Diavatopoulos DA, Reading PC, Brown LE, Rogers KL, Strugnell RA, Wijburg OL. 2011. Using bioluminescent imaging to investigate synergism between *Streptococcus pneumoniae* and influenza A virus in infant mice. J Vis Exp 2011:2357. doi:10.3791/2357. PMC3169275 21525841

[86] Alam FM, Turner CE, Smith K, Wiles S, Sriskandan S. 2013. Inactivation of the CovR/S virulence regulator impairs infection in an improved murine model of *Streptococcus pyogenes* nasopharyngeal infection. PLoS One 8 doi:10.1371/annotation/1144e132-9e69-47bb-8e65-1414dbb01db7. PMC3636223 23637876

[87] Van Elden LJR, Nijhuis M, Schipper P, Schuurman R, Van Loon AM. 2001. Simultaneous detection of influenza viruses A and B using real-time quantitative PCR. J Clin Microbiol 39:196–200. doi:10.1128/JCM.39.1.196-200.2001. 11136770 PMC87701

[88] McAuley JL, Hornung F, Boyd KL, Smith AM, McKeon R, Bennink J, Yewdell JW, McCullers JA. 2007. Expression of the 1918 influenza A virus PB1-F2 enhances the pathogenesis of viral and secondary bacterial pneumonia. Cell Host Microbe 2:240–249. doi:10.1016/j.chom.2007.09.001. 18005742 PMC2083255

[89] McAuley JL, Zhang K, McCullers JA. 2010. The effects of influenza A virus PB1-F2 protein on polymerase activity are strain specific and do not impact pathogenesis. J Virol 84:558–564. doi:10.1128/JVI.01785-09. 19828614 PMC2798424

[90] National Health and Medical Research Council. 2013. Australian code for the care and use of animals for scientific purposes, 8th ed. National Health and Medical Research Council, Canberra, Australia. https://www.nhmrc.gov.au/about-us/publications/australian-code-care-and-use-animals-scientific-purposes#block-views-block-file-attachments-content-block-1.

